# Influence of Cesarean Section on Postpartum Fertility and Dysmenorrhea: A Retrospective Cohort Study in Japan

**DOI:** 10.1089/whr.2023.0109

**Published:** 2024-01-12

**Authors:** Mizuki Ohashi, Shunichiro Tsuji, Kyoko Kasahara, Ryoko Oe, Yumiko Tateoka, Takashi Murakami

**Affiliations:** ^1^ Department of Obstetrics and Gynecology, Shiga University of Medical Science, Otsu, Shiga, Japan.; ^2^ NCD Epidemiology Research Center, Shiga University of Medical Science, Otsu, Shiga, Japan.; ^3^ Department of Clinical Nursing, Shiga University of Medical Science, Otsu, Shiga, Japan.

**Keywords:** cesarean section, dysmenorrhea, maternal fertility, retrospective study, secondary infertility

## Abstract

**Objective:**

To investigate the association between cesarean section (CS) and postpartum fertility and dysmenorrhea using data from a Japanese insurance registry.

**Methods:**

This retrospective cohort study used a data set of patients registered between 2007 and 2021 in an insurance registry comprising specific employee-based health insurance companies in Japan. Of those data sets, we included data from participants who had their first recorded childbirth between 2014 and 2018. The exclusion criteria were any prior deliveries, dysmenorrhea, or complications that would affect the next pregnancy or postpartum dysmenorrhea since 2007. The occurrence of subsequent childbirth and postpartum dysmenorrhea until 2021 was compared between the CS and vaginal delivery (VD) groups using the log-rank test and Cox proportional hazards model with stratification according to age and age matching.

**Results:**

This study included 25,984 (5,926 after age matching) and 5,926 participants in the VD and CS groups, respectively. After age matching, the rate of subsequent childbirth was 18.3% and 16.3%, and the rate of postpartum dysmenorrhea was 6.5% and 7.8% in the VD and CS groups, respectively. There were fewer subsequent childbirths in the CS group than in the VD group after age matching in the stratified Cox proportional hazards model (hazard ratio [HR] 95% confidence interval [CI]: 0.86 [0.79–0.94]). The CS group had a significantly higher risk of dysmenorrhea (HR [95% CI]: 1.18 [1.03–1.36]).

**Conclusions:**

Although confounding might be existing, our study suggests that CS might be associated with decreased postpartum fertility and increased dysmenorrhea. The medical indications for CS should be carefully determined; post-CS women should be meticulously followed up.

## Introduction

Cesarean section (CS) is an essential procedure that saves the lives of both the mother and the child. CS rates have increased worldwide, and Japan had a CS rate of 18.6% in 2014, despite the World Health Organization stating that CS rates >10% are not associated with a reduction in maternal and newborn mortality.^1,2^

Some reports have suggested that CS may reduce subsequent fertility at higher rates than that observed in women who give birth *via* vaginal delivery (VD),^3–6^ whereas another study has indicated that CS has only a slight impact on future fertility.^7^ To the best of our knowledge, no reports from Japan have been published on this subject.

Dysmenorrhea, especially secondary dysmenorrhea, has a potential association with infertility. Secondary dysmenorrhea is caused by endometriosis, uterine fibroids, and other diseases or inflammation in women's reproductive organs. These conditions lead to infertility due to failure of implantation. A previous CS may cause uterine cesarean scar defects (CSD), which lead to cesarean scar disorder (CSDi) characterized by symptoms such as secondary dysmenorrhea and infertility probably due to chronic inflammation *in utero*.^8–12^ A previous study showed a significant association between CSD and infertility.^13^

Therefore, the incidence of postpartum dysmenorrhea may be influenced by the mode of delivery. However, in a previous meta-analysis of the long-term outcomes of CS, only one randomized controlled trial indicated dysmenorrhea, and the patients were followed up for 2 years without any significant difference.^14,15^ There has been no long-term comparison of the incidence of postpartum dysmenorrhea between patients from Asian countries who underwent CS and those who underwent VD. Therefore, understanding the influence of CS on women's postpartum health is important; especially, the long-term influence of >2 years should be investigated as the average age difference between the first and second children in Japan is 1.8–2.0 years.^16^ Moreover, there is no large-scale cohort study on this topic.

We hypothesized that CS has an adverse influence on postpartum fertility and dysmenorrhea, and this retrospective cohort study aimed to determine the association between CS and postpartum fertility and dysmenorrhea using a large-scale data set from a Japanese insurance registry.

## Materials and Methods

### Study population

This retrospective cohort study used a data set obtained from patient health insurance claims of employee-based insurance in Japan, which was provided by the Japan Medical Data Center database (JMDC Inc., Tokyo, Japan). All patients enrolled in specific employee-based health insurance companies under contracts with JMDC were included in the data set. We could access all insurance-covered procedures, medications, and the dates of these treatments for registered patients between 2007 and 2021. In addition, data of the patient's family members registered under the same insurance could be accessed.

Among all data sets, we extracted a data set that linked birthing-parent and child data (*n* = 172,730), and we limited the data set to participants for whom no prior delivery had been recorded between 2007 and 2013 to exclude multiparous participants as far as possible. Index delivery was defined as the first recorded childbirth between 2014 and 2018 in our data set, and we limited the data set to participants who had >2 years of available data before the index delivery. This group included 47,755 participants who had their first recorded childbirth between 2014 and 2018. The participants were followed up until the end of 2021.

**FIG. 1.**
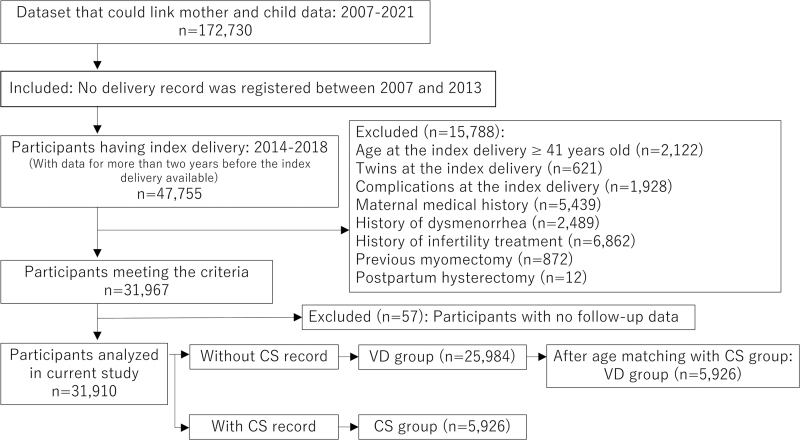
Flow chart of study participants selection. The data set was limited to participants who had no record of prior delivery in our data set between 2007 and 2013. Index delivery was defined as the first childbirth recorded in the data set between 2014 and 2018. Medical histories and complications were identified using the Tenth Revision of the International Classification of Diseases codes. Japanese standardized procedure codes were used to search for procedures, such as CS or other surgeries. Data on the use of medications for infertility treatment were retrieved using the Anatomical Therapeutic Chemical Classification System. The CS group was defined based on the CS record (Japanese standardized procedure code: K898). CS, cesarean section; VD, vaginal delivery.

We excluded participants who were ≥41 years old at index delivery (*n* = 2122), delivered twins (two babies delivered by the same person in the same year and month) at index delivery (*n* = 621), had complications at index delivery (*n* = 1,928), had a medical history associated with infertility (*n* = 5,439), had a history of dysmenorrhea (*n* = 2,489), underwent infertility treatment (*n* = 6,862), had a previous myomectomy (*n* = 872), and underwent a hysterectomy postpartum (*n* = 12). We further excluded participants with no follow-up data (*n* = 57). The remaining 31,910 participants were included in the study. The VD group included 25,984 participants, and the CS group included 5,926 participants (Fig. 1).

The data are not publicly available due to the contract with JMDC.

### Definitions

Medical histories and complications were identified using the Tenth Revision of the International Classification of Diseases (ICD-10) codes. Japanese standardized procedure codes were used to search for procedures, such as CS or other surgeries. Data on the use of medications for infertility treatment were retrieved using the Anatomical Therapeutic Chemical Classification System.

To identify childbirth, we extracted registered newborns' birth years and months from their insurance records. Complications at index delivery were extracted according to diagnoses made between 6 months and 1 month before the index delivery, such as hypertensive disorders of pregnancy (ICD-10 codes: O10–O16), gestational diabetes mellitus (ICD-10 code: O24), placental anomalies (ICD-10 code: O44), and early abruption of the normal placenta (ICD-10 code: O45). Maternal medical history that could be associated with infertility was extracted based on diagnoses applied between 2 years and 6 months before the index delivery, such as hypertension (ICD-10 codes: I10–I15), diabetes (ICD-10 codes: E10–E14), and asthma (ICD-10 code: J45).

These factors were defined as complications because these have already been reported as risk factors for infertility in previous studies.^17–21^ History of dysmenorrhea was defined as a history of dysmenorrhea (ICD-10 codes: N944–N946) and endometriosis (ICD-10 code: N80) diagnosed between 2 years and 6 months before the index delivery. A history of infertility treatment was defined as the use of clomiphene, cyclofenil, or human menopausal gonadotrophin, which had been administered between 2 years and 6 months before the index delivery.

Previous myomectomy was defined by the Japanese standard procedure code (K872), which was performed between 2 years and 6 months before the index delivery. Postpartum hysterectomy was defined using the Japanese standard procedure codes (K877 and K877-2), which were performed after delivery. CS was defined using the Japanese standard procedure code (K898), which was performed in the same year and month as childbirth. No distinction was made between a planned and emergency CS in the study.

### Statistical analysis

We used survival analysis methods to estimate the influence of CS on the time to subsequent childbirth and development of dysmenorrhea in participants without any complications or medical history that may be risk factors for infertility. We compared the occurrence of subsequent childbirth and postpartum dysmenorrhea between the CS and VD groups using the log-rank test, hazard ratio (HR), and 95% confidence interval (CI) using the Cox proportional hazards model with and without stratification according to age and the Fine and Gray model with dropout due to death as a competing risk.

Time to subsequent childbirth was defined as the date (year and month) of the registered birth of the next child. Censoring events were defined as withdrawal from insurance, reaching 7 years after the index childbirth, or the end of the study period (December 2021), whichever came first. Concerning postpartum dysmenorrhea, the time to the development of dysmenorrhea was defined as the date of newly registered ICD-10 codes for dysmenorrhea (N944–N946) or endometriosis (N80) after index childbirth. Censoring events were defined as withdrawal from insurance, reaching 7 years after the index childbirth, the next pregnancy, which was determined 6 months before the subsequent childbirth, or the end of the study period (December 2021), whichever came first.

**FIG. 2.**
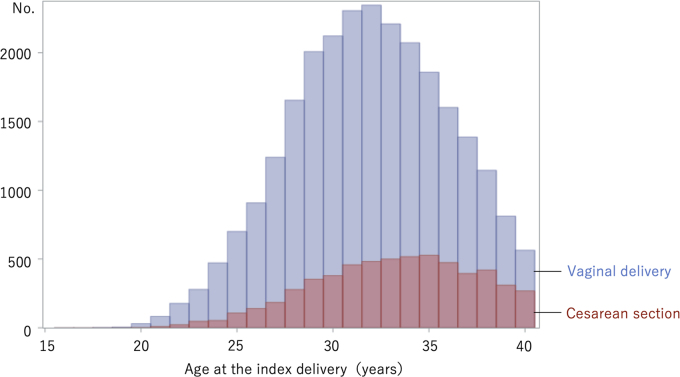
Distribution of age at the index delivery in the VD and the CS groups. The number of participants is shown according to the age at index delivery. The VD group included 25,984 participants, and the CS group included 5,926 participants.

Age distribution differed between the VD and CS groups, and the subsequent childbirth rate varied according to the age at first delivery, as shown in Figure 2 and Supplementary Figure S1. Therefore, we performed the same analyses after age matching so that the number of participants and their age distribution in the VD and CS groups were the same.

All statistical analyses were performed using SAS version 9.4 (SAS Institute Inc., Cary, NC, USA). Two-sided tests with *p* < 0.05 were deemed statistically significant.

This study was conducted following proper guidelines, such as the Japanese Ethical Guidelines for Medical and Biological Research Involving Human Subjects. Informed consent was not required from the participants due to the use of deidentified data. The study protocol was approved by the appropriate institutional review board of the Shiga University of Medical Science (R2022-187).

## Results

The study included 31,910 participants aged 16–40 years at the time of index childbirth. The VD group included 25,984 participants and the CS group included 5,926 participants, representing 18.6% of all participants. The average follow-up time was 54.8 months. The proportion of subsequent childbirth was 5,468 (21.0%) and 965 (16.3%) in the VD and CS groups, respectively.

The proportion of postpartum dysmenorrhea was 1,806 (7.0%) and 459 (7.8%) in the VD and CS groups, respectively. After age matching, there were 5,926 participants in each group, and the proportions of subsequent childbirth and postpartum dysmenorrhea in the VD group were 1,085 (18.3%) and 382 (6.5%), respectively. Only 11 (9 and 2 in the VD and CS groups, respectively) participants were censored because of withdrawal from insurance due to death.

**FIG. 3.**
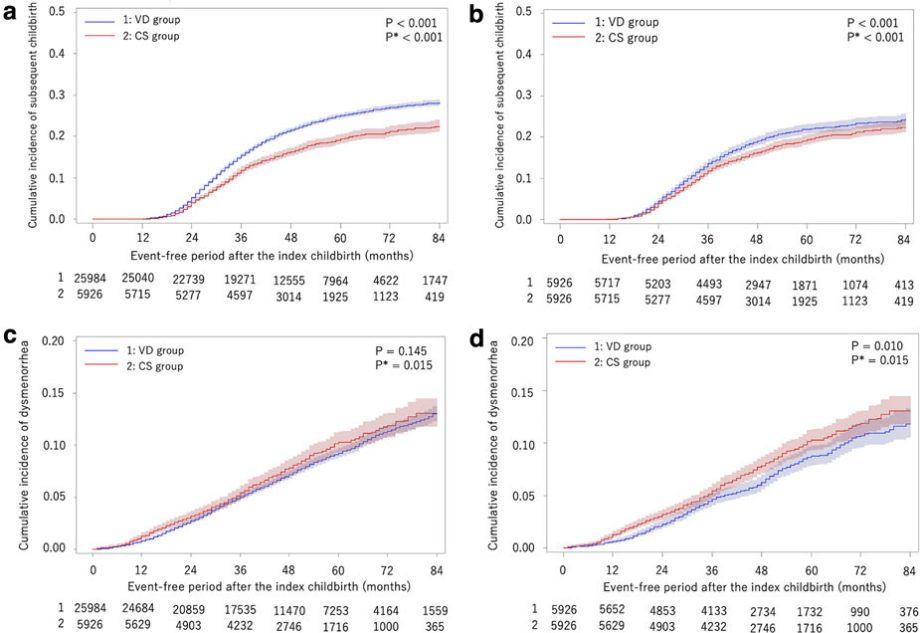
Kaplan–Meier curves for the incidence of subsequent childbirth and dysmenorrhea. Kaplan–Meier curves for the incidence of subsequent childbirth and dysmenorrhea and *p*-values for log-rank tests. **(a)** Results for subsequent childbirth before age matching, **(b)** results for subsequent childbirth after age matching, **(c)** results for dysmenorrhea before age matching, and **(d)** results for dysmenorrhea after age matching. The VD group (1) included 25,984 participants and the CS group (2) included 5,926 participants. After age matching, the VD group included 5,926 participants similar to that in the CS group. The number of people at risk is shown at the bottom of the table. *p*, *p*-Value for the log-rank test; p*, *p*-Value for the log-rank test stratified by age.

The results for subsequent childbirths before and after age matching are shown in Figure 3 and Table 1. The Kaplan–Meier curves demonstrated that participants in the CS group were less likely to have a subsequent childbirth than those in the VD group, and this association was significant with and without stratification according to age and before and after age matching.

**Table 1.** Hazard Ratios and 95% Confidence Intervals for the Incidence of Subsequent Childbirth and Dysmenorrhea

Outcome	Statistical method	HR (95% CI) for CS vs. VD	
		Before age matching (***n*** = 31,910)	After age matching (***n*** = 11,852)
Subsequent childbirth	Cox proportional hazards model	0.74 (0.70–0.79)	0.87 (0.80–0.95)
	Stratified Cox proportional hazards model according to age	0.86 (0.80–0.91)	0.86 (0.79–0.94)
	Fine and Gray model	0.86 (0.80–0.91)	0.86 (0.79–0.94)
Postpartum dysmenorrhea	Cox proportional hazards model	1.08 (0.97–1.20)	1.20 (1.04–1.37)
	Stratified Cox proportional hazards model according to age	1.14 (1.03–1.26)	1.18 (1.03–1.36)
	Fine and Gray model	1.14 (1.03–1.26)	1.18 (1.03–1.36)

HRs and 95% CIs for the CS group versus the VD group were estimated using the Cox proportional hazards model, stratified Cox proportional hazards model according to age, and the Fine and Gray model with dropout due to death as a competing risk. The VD group included 25,984 participants and CS group included 5,926 participants. After age matching, the VD group included 5,926 participants, the same as the CS group.

CI, confidence interval; CS, cesarean section; HR, hazard ratio; VD, vaginal delivery.

The HRs (95% CIs) for the CS group versus the VD group before and after age matching were 0.74 (0.70–0.79) and 0.87 (0.80–0.95) for the Cox proportional hazards model, 0.86 (0.80–0.91) and 0.86 (0.79–0.94) for the Cox proportional hazards model stratified according to age, and 0.86 (0.80–0.91) and 0.86 (0.79–0.94) in the Fine and Gray model, respectively. Subsequent childbirths decreased in the CS group, and this effect was attenuated but remained significant after age matching.

The results of the development of postpartum dysmenorrhea before and after age matching are shown in Figure 3 and Table 1. Kaplan–Meier curves demonstrated that participants in the CS group were more likely to develop postpartum dysmenorrhea than those in the VD group, and this association was significant according to the log-rank test stratified according to age before and after age matching.

The HRs (95% CIs) for the CS group versus the VD group before and after age matching were 1.08 (0.97–1.20) and 1.20 (1.04–1.37) in the Cox proportional hazards model, 1.14 (1.03–1.26) and 1.18 (1.03–1.36) in the Cox proportional hazards model stratified according to age, and 1.14 (1.03–1.26) and 1.18 (1.03–1.36) in the Fine and Gray model, respectively. Postpartum dysmenorrhea increased in the CS group and this effect remained significant after age matching.

In our study, dropouts due to death did not play a significant role because the results from the Fine and Gray models did not differ from the other results.

## Discussion

In this study, the number of subsequent childbirths was significantly lower in the CS group than in the VD group. The incidence of postpartum dysmenorrhea was significantly higher in the CS group than in the VD group. These associations could have been confounded by maternal age; however, they remained significant in both the log-rank test and Cox proportional hazards model, which were stratified according to age. Furthermore, these associations remained significant even after matching for age.

The influence of CS on subsequent childbirth in our study was mostly consistent with that demonstrated in previous reports.^3–6^ Clinical and social factors during pregnancy and the intrapartum period leading to CS have a greater effect on future fertility than the CS itself, because the effect of elective CS for breech on subsequent fertility is small, whereas the effect of elective CS for other indications is large.^7^ However, we observed a significant negative association of CS on subsequent childbirth even after excluding potential risk factors for infertility, including maternal complications and medical history.

Our results indicate that CS was associated with both decreased postpartum fertility and increased dysmenorrhea, although it is difficult to determine whether dysmenorrhea causes infertility. However, some evidence suggests that dysmenorrhea may be related to infertility and that this relationship may be caused by CS.

In women who undergo CS, CSD may lead to CSDi, which is a potential cause of infertility and dysmenorrhea.^8–11^ CSDs are found in most postpartum women who deliver *via* CS,^22,23^ and their prevalence can differ depending on the procedure.^23^ Abnormal subendometrial contractions have been observed in women with CSD and may cause dysmenorrhea and abnormal uterine bleeding.^9,10^ Chronic inflammation has also been detected *in utero* in women with CSD.^24,25^

Women with dysmenorrhea have a higher incidence of endometriosis, possibly because the backflow of chronically inflamed menstrual blood into the pelvis induces endometriosis and secondary dysmenorrhea. Moreover, chronic inflammation of the uterus and pelvis may contribute to infertility. The risk of recurrent endometriosis, which causes infertility, is also higher after CS.^26^ Therefore, it is important to avoid induced CS and reduce the incidence of dysmenorrhea, which may lead to infertility.

As another possibility for those results, the following hypothesis may be also considered. Women with emergency CS are at increased risk of a traumatic experience due to which they postpone the next pregnancy. The women in the VD group are more prone to become pregnant, and they do not suffer from dysmenorrhea during their pregnancy and lactation period. These combined factors may have led to the present results.

One strength of our study is its large sample size, which is important for assessing the nationwide situation in Japan. In addition, the follow-up period was sufficient to evaluate subsequent childbirths. Nonetheless, our study had some limitations. We did not include participants for whom no deliveries were recorded between 2007 and 2013 in our data set. However, we did not completely exclude multiparous participants, as participants with childbirth intervals longer than 8 years might have been included in our analyses.

Nevertheless, the proportion of such participants is expected to be small since the average age difference between first and second children in Japan was 1.8–2.0 years between 2014 and 2018 according to a report from Japanese vital statistics.^16^ Moreover, we had limitations in collecting information that may have influenced fertility and dysmenorrhea, such as obesity, because we used a health insurance-based registered data set. However, we tried to minimize bias by excluding data, such as maternal complications, that may have affected the outcomes of this study. The participants are limited to people registered by employee-based insurance; however, the generalizability of our result is high as this data set covers multifacility and regions all over Japan.

## Conclusion

Our findings suggest that CS might be associated with postpartum infertility and dysmenorrhea. Therefore, the medical indications for CS should be carefully determined, considering its potential impact on postpartum health. Meticulous follow-up of post-CS women is also important to prevent infertility and loss of quality of life due to dysmenorrhea.

## Supplementary Material

Supplemental data

## Abbreviations Used

CI: confidence interval
CS: cesarean section
CSD: cesarean scar defects
CSDi: cesarean scar disorder
HR: hazard ratio
ICD-10: Tenth Revision of the International Classification of Diseases
VD: vaginal delivery
