# Supplementary material for: Influence of Cesarean Section on Postpartum Fertility and Dysmenorrhea: A Retrospective Cohort Study in Japan
Source: Womens Health Rep (New Rochelle). 2024 Jan 12;5(1):22–9. doi: 10.1089/whr.2023.0109 (PMC10797175; doi:10.1089/whr.2023.0109)

**Figure Legends:**

**Supplementary figure 1. Kaplan–Meier curves for the incidence of subsequent childbirth by age group**

Kaplan–Meier curves for the incidence of subsequent childbirths among all participants are shown by age group: (1) ≤29 years, (2) between 30–34 years, or (3) ≥35 years. The numbers of people at risk are shown at the bottom of the plot.
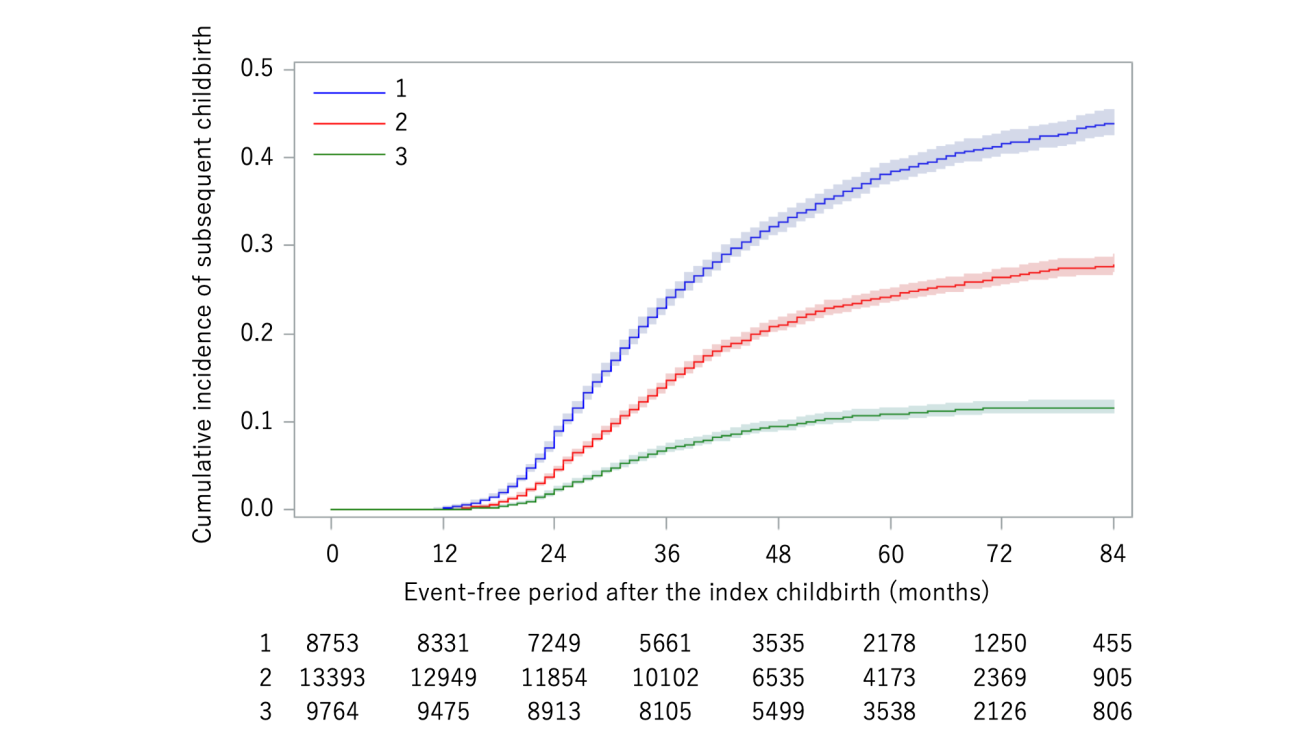

Supplement: Supplemental data [file Suppl_FigureS1.docx]
